# Betrayal Not Compromise

**Published:** 1919-04-26

**Authors:** 


### THE MATRONS' AND SISTERS' DEPARTMENT.

### BETRAYAL NOT COMPROMISE.

THERE has been printed and issued the Nurses' Registration Bill as amended in Standing Committee E, and engineered by the party which has for years fostered disunion in the nursing profession. They were aided by the Government representatives, under the camouflage of "compromise" which was no compromise. Mr. Lyle exposed this position, which amounts to a betrayal of the interests of the working nurse, and a travesty of justice so far as the great bulk of the members of the nursing profession are concerned. The Bill as it stands, if allowed to pass uncontested, would give the control and influence to persons who have had little or nothing to do with the training, education, and creation of British nurses as they exist to-day. The promoters of the College Bill include women who have done much for the Empire and the world throughout the war, and who represent some of the brightest minds and ablest heads in the nursing profession in these islands. It is not credible that the members of the present House of Commons will pass the Central Committee's Bill as it stands to-day, when they are brought to realise the position that has been created by Standing Committee E. This inflicts gross injustices and wanton insults under Clause 4 to the majority of the nursing profession, the real leaders of nursing in this country, and the nurse training-schools within its confines. The creators of disunion, some of whom have shown in the past the undue attraction which money may possess, have by clever tactics seized the control of nurse registration. As the Bill at present stands a party of discordant, personal, axe-grinding interests may dominate the General Nursing Council, and possess the power to standardise the training, the examination and the registration of British nurses for the first two years of the existence of registration. This has been accomplished by giving representation to certain societies and associations with high-sounding names, mostly originating with the skeleton army party, which have recently been affiliated with the Royal British Nurses' Association. There are seven of these societies, and not only has the Royal British Nurses' Association been given four votes—an identical number to that given to the College of Nursing with its 13,500 registered nurses—but seven representatives are added to the party of disunion by giving one to each of these societies, although they are already represented by affiliation with the Royal British Nurses' Association and so practically included in it.

This monstrous iniquity was made possible at the last minute by the intervention of Colonel Nathan Raw, who, instead of supporting the College by his act, threw it to the wolves. This action and other proceedings of the gentleman in question have created justifiable anger throughout the majority of the nursing profession, and he can no longer be considered a friend, much less a representative, of the best interests of the nursing profession or of the College of Nursing. Had Colonel Raw not intervened, Mr. Lyle's very able speech exposing the injustices perpetrated, there is no reasonable doubt, would have carried the majority of the Committee. The consequences of the proceedings in question are so shameless, in fact, that they cannot fail to cause justifiable indignation on the part of every independent member of the House of Commons when he realises the actual position in nursing affairs which the Bill, as amended by Standing Committee E, creates. In this connection we refer our readers to an article by "Ierne" on page 88, who exposes the non-representative character and shams of two Irish associations, which are in reality one, each of whose representatives is made a member of the Nursing Council. Further, the Bill as it stands supersedes Scotland and deprives the majority of Scotch nurses and training-schools of representation, an act of impertinence which is likely to bring condign punishment upon those who are responsible for this attempted outrage upon Scotland, its training-schools, its nurses, and people.

Such is the position which skilful engineering and intrigue have created in Standing Committee E. Every nurse who is proud of her profession and realises, as the majority of nurses do, the injury and insult she has suffered for so many years from several of the parties who have united to perpetrate this iniquity, may be assured that the sympathies of the whole nation will go out to the nursing profession at this crisis, and Members of Parliament are likely to experience an outbreak of indignation and a widespread demand for justice for the nurse and the nurse training-schools, which should not fail speedily to adjust the balance and substitute a Registration Bill which gives justice to all interests, and protection everywhere for the working nurse. Mr. Lyle strenuously and justly insisted that some effort should have been made by the President of the Local Government Board to go into the relative strength and status, the standing, power, and position of the various societies claiming to direct through their representatives the nursing profession. Had he done so the plot which has so far succeeded must have failed. Some such course must now be taken if a Registration Bill is ever to pass the Houses of Parliament and secure the Royal assent.

### The Unexpected Happens.

Two unforeseen alterations to the Bill are the provision of a separate Register of Children's Nurses and the introduction of proportional representation. It is hardly possible that members of the general public should understand the danger

**Betrayal Not Compromise**—(*continued*).

of registering nurses under a training carried out from start to finish among young children. Had the matter been left to the Nursing Council to arrange some safeguards could have been introduced. As it stands, the clause introduced in Committee makes it possible for a young woman whose experience has been entirely among little children to be mistaken for a fully-qualified nurse and to be entrusted with difficult medical and surgical cases demanding skill and knowledge she has never had a chance of acquiring. The matter is one of great public importance which may yet be put right when the Bill comes before Parliament. It is extraordinary that the proposal to form such a Register should proceed from a medical man, and be supported by his *confrères*. None are likely to regret this hasty step more than doctors should the clause be approved in the end.

We confess ourselves satisfied with the sudden decision of the Committee to give proportional representation to nurses under the Bill. What nurses have to dread more than anything else in their efforts to get represented on the Provisional and General Councils is the Caucus system, under which votes can be manipulated at will by whoever happens to hold the first Committee on a tight rein. Proportional Representation is a system of voting under which each elector records not only his vote for the candidate he prefers, but also his second, third, fourth, or fifth choice, should his first-named candidate have more votes than are needed to secure his election, or so few votes that he is at the bottom of the poll. In either of these cases the votes recorded are lost under the ordinary system. Under Proportional Representation they are transferred to the candidate numbered by the voter next in order, and instead of being lost are turned to good account precisely as the voter would wish. Thus, suppose among the candidates there is one world-famed doctor who has an enormous preponderance of votes, only sufficient will be credited to him to ensure his election. The other votes standing to his name will go to the people marked as second choice by those who voted for him. In this way nurses can exercise a real influence in appointing their representatives. Custom or clever suggestion may dictate the first choice. But no one can calculate or control the second or third choice. The effect is to ensure that all currents of opinion are represented and not merely one party.

